# PBPK Modeling Investigation of IVIVE–Observed Pharmacokinetic Divergence for VU0467319: A Mechanistic Refinement Workflow for Low-Solubility Compounds

**DOI:** 10.3390/pharmaceutics18101254

**Published:** 2026-10-02

**Authors:** Akinwunmi A. Abisogun, Srinivasan Krishnan, Craig W. Lindsley, Olivier Boutaud

**Affiliations:** 1 Warren Center for Neuroscience Drug Discovery, Vanderbilt University, Nashville, TN 37232, USA; akinwunmi.abisogun@vanderbilt.edu (A.A.A.); srinivasan.krishnan@vanderbilt.edu (S.K.);; 2 Vanderbilt Institute for Therapeutic Advances, Vanderbilt University, Nashville, TN 37232, USA; 3 Department of Pharmacology, School of Medicine, Vanderbilt University, Nashville, TN 37232, USA

**Keywords:** PBPK, physiologically based pharmacokinetics, IVIVE, dissolution-limited absorption, Simcyp, VU0467319, M1 muscarinic positive allosteric modulator, low-solubility compounds, HLM/HH disconnect, pharmacokinetic modeling

## Abstract

**Background/Objectives:** VU0467319 (VU319), an M1 muscarinic positive allosteric modulator evaluated in Phase 1 studies, exhibits subproportional dose-exposure scaling typical of poorly soluble compounds. This study applies physiologically based pharmacokinetic (PBPK) modeling to investigate the sources of divergence between IVIVE-based predictions and observed single ascending dose (SAD) clinical pharmacokinetics. **Methods:** A whole-body PBPK model was developed in Simcyp Simulator V24 using in vitro ADME and physicochemical inputs. Clinical pharmacokinetic data were used for sequential, hypothesis-driven model refinement of distribution, clearance, and absorption parameters. Model performance was evaluated at 60–600 mg fasted using virtual populations demographically matched to their corresponding clinical cohort. **Results:** A K_p_ scalar of 0.125 recovered model V_ss_ within the observed V_z_/F bound; a 10.7-fold downward CL_int_ correction (32.2 to 3 µL/min/mg) reconciled clearance; and a 1 µm particle radius recovered AUC_0–∞_ within 6% across 60–240 mg. The model met predefined performance thresholds across the four independent verification doses (geometric mean fold error 1.45 for AUC and 1.56 for C_max_; three of four doses within 2-fold), with progressive underprediction at the highest doses (≥400 mg). Supersaturation-ratio analysis indicated that, within the model, sustained supersaturation was unlikely to explain the high-dose underprediction. The refined model reproduced the observed sub-proportional dose-exposure relationship through 240 mg, but underpredicted exposure at 400 and 600 mg. **Conclusions:** Structured PBPK refinement identified dissolution-limited absorption as the most likely primary contributor to IVIVE–observed divergence for VU319, with secondary contributions from clearance and distribution. These model-derived mechanisms remain hypotheses that require experimental confirmation. The workflow may provide a structured refinement framework for other low-solubility compounds where IVIVE assumptions diverge from observed pharmacokinetics.

## 1. Introduction

Physiologically based pharmacokinetic (PBPK) modeling is widely employed to predict clinical pharmacokinetics and support regulatory submissions. PBPK models integrate physiologically relevant compartmental frameworks with compound-specific in vitro-in vivo extrapolation (IVIVE) of absorption, distribution, metabolism, and excretion parameters [1,2]. IVIVE differs from the in vitro-in vivo correlation (IVIVC) and evaluates the relationship between in vitro ADME properties and observed in vivo pharmacokinetics. In contrast, IVIVE uses experimentally determined in vitro ADME parameters to predict in vivo pharmacokinetics through physiological scaling. For compounds with predictable pharmacokinetic behavior, this bottom-up approach can adequately recapitulate observed clinical pharmacokinetics using in vitro-derived inputs alone. However, for compounds with challenging physicochemical characteristics, conventional IVIVE assumptions often fail to accurately capture observed clinical pharmacokinetics, thereby necessitating additional mechanistic refinement. In such cases, PBPK can be used as an investigative tool for identifying which experimentally informed assumptions contribute to the IVIVE–observed discrepancy, rather than as a prospectively validated predictive model [3,4].

Three major categories of IVIVE divergence have been consistently described in the literature. First, hepatic clearance predicted from human liver microsomal intrinsic clearance (HLM CL_int_) may differ substantially from observed in vivo clearance, particularly for lipophilic compounds in which hepatic uptake becomes rate-limiting for intracellular metabolism [3,5]. Second, mechanistic tissue partitioning approaches, including the Poulin & Theil and Rodgers & Rowland methods, may inaccurately predict tissue distribution for highly lipophilic compounds or for compounds that fall outside the ionization assumptions underlying these models [6,7]. Third, default Advanced Dissolution, Absorption, and Metabolism (ADAM) model inputs may inadequately capture in vivo dissolution characteristics for poorly soluble compounds, frequently resulting in underprediction of exposure at higher doses [4,8].

When IVIVE diverges from observed pharmacokinetics, PBPK functions less as a prediction engine and more as an investigative framework. Sequential refinement of individual parameter categories, guided by mechanistic hypotheses with literature precedent, can identify the mechanisms most likely responsible for observed divergence [3,4,9]. Importantly, these refinements are used to evaluate which IVIVE assumptions may account for the observed discrepancy, rather than to replace experimentally derived inputs or independent validation.

VU0467319 (VU319) is a selective M_1_ muscarinic positive allosteric modulator developed as a candidate cognitive enhancer for Alzheimer’s disease [10]. The compound progressed through IND-enabling studies and subsequently completed a Phase 1 single ascending dose (SAD) in healthy volunteers [11]. The SAD study evaluated five fasted dose cohorts (60, 120, 240, 400, and 600 mg) and demonstrated sub-proportional increases in exposure across the dose range, with a mean terminal half-life of 30–55 h and oral clearance values ranging from 1.73–3.42 L/h. VU319 is highly lipophilic (logP = 4.18), weakly basic (monoprotic; conjugate-acid pK_a_ = 1.4; essentially unionized as the neutral free base at physiological pH), and exhibits low plasma unbound fraction (*f*_u_ = 0.034) and low aqueous solubility, properties collectively suggestive of Biopharmaceutics Classification System (BCS) Class II behavior.

This study applies PBPK modeling to investigate divergence between IVIVE-based predictions and observed VU319 SAD pharmacokinetics. Three sequential hypotheses were evaluated: (H1) Method 1 tissue distribution does not accurately recover observed V_ss_ for the compound’s lipophilicity and ionization profile; (H2) microsomal CL_int_ scaled using standard IVIVE methods does not recover observed in vivo clearance; and (H3) default absorption parameters do not adequately capture the dissolution kinetics governing in vivo exposure. The objectives were to identify the mechanistic source most likely responsible for the observed divergence, establish a systematic refinement workflow applicable to other poorly soluble compounds, and generate testable mechanistic hypotheses to inform subsequent formulation and multiple-dose studies.

## 2. Materials and Methods

### 2.1. Software

Simulations were conducted in Simcyp Simulator V24 (Certara, Sheffield, UK) using the Healthy Volunteers population under fasted single-dose SAD conditions. Numerical integration was performed using the native Sundials CVODE solver as implemented in Simcyp Simulator V24 with relative and absolute tolerances of 1 × 10^−7^ and 1 × 10^−9^, respectively. Data processing and figure generation were performed using R Statistical Software Version 4.5.3 (2026-03-11) (R Foundation for Statistical Computing, Vienna, Austria) via the RStudio Integrated Development Environment (IDE) Version 2026.01.2+418 (Posit Software, PBC, Boston, MA, USA).

### 2.2. Model Structure and Assumptions

**Figure 1:**
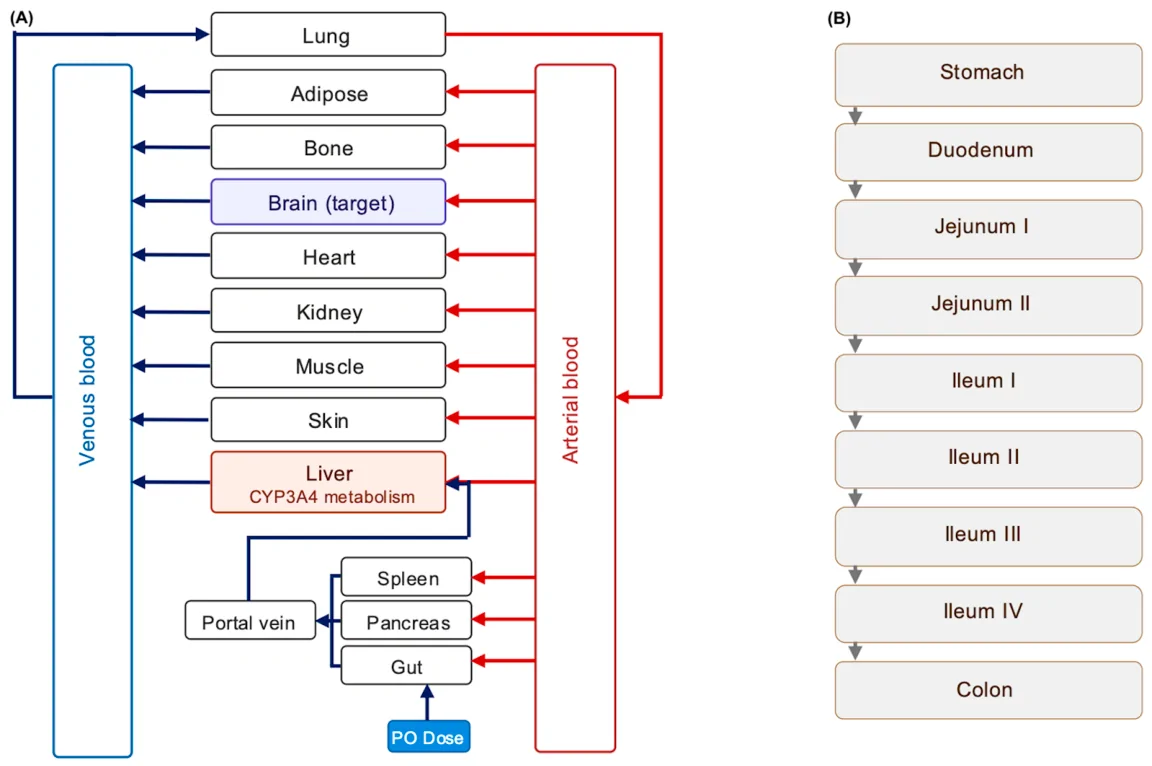
Graphical representation of the VU319 PBPK model implemented in Simcyp. The model consists of a whole-body, perfusion-limited PBPK structure (**A**) coupled to an Advanced Dissolution, Absorption and Metabolism (ADAM) gastrointestinal absorption model (**B**). Organ compartments are connected through arterial (red) and venous (blue) circulation and linked by organ-specific blood flows and tissue-to-plasma partition coefficients (K_p_). Tissue distribution was modeled using the Poulin & Theil method with a global K_p_ scalar of 0.125 applied during refinement. Hepatic elimination was modeled as CYP3A4-mediated metabolism within the liver compartment using a calibrated intrinsic clearance (CL_int_ = 3 µL/min/mg microsomal protein; *f*_u,mic_ = 0.41). Renal clearance was modeled as negligible, consistent with the reported clinical disposition profile. Splanchnic compartments drain through the portal vein into the liver to represent first-pass extraction. Oral absorption incorporated pH-dependent solubility, particle-based dissolution, precipitation kinetics, segmental permeability, and gastrointestinal transit. Dissolution refinement used a calibrated particle radius of 1 µm.

A full-body, perfusion-limited PBPK model was implemented with organ-specific blood flows and tissue-to-plasma partition coefficients (K_p_) under well-stirred tissue assumptions (Figure 1). Tissue distribution was modeled through passive diffusion without explicit transporter-mediated uptake or efflux.

Oral absorption was simulated using the ADAM model with the immediate-release Diffusion Layer Model (IR:DLM), in which the drug was administered as a solid and dissolution was calculated from a monodispersed particle population using the particle population balance approach. Particles were described by a monodisperse radius (10 µm at baseline; calibrated to 1 µm), a particle density of 1.2 g/mL, a predicted diffusion coefficient (4.17 × 10^−4^ cm^2^/min), and a diffusion layer thickness calculated by the Hintz–Johnson method (30 µm cut-off). The particle population balance radius rate cap was set to 2000 µm/h for all simulations. Solubility was defined by the pH-solubility profile (FaSSGF, 0.05 mg/mL at pH 1.6; FaSSIF, 0.00532 mg/mL at pH 6.5; intrinsic solubility, 0.00409 mg/mL, predicted), with log-linear interpolation between pH values, and bile micelle-mediated solubilization was predicted from logP. Precipitation was described by a first-order model using the Simcyp default precipitation rate constant (4 h^−1^) and critical supersaturation ratio (10). Gastrointestinal transit was described by the segregated transit time model using default fasted-state values, including a gastric mean residence time of 0.27 h and a small-intestinal mean residence time of 3.4 h, with a fluid intake of 250 mL and default fasted-state intestinal fluid volumes and bile salt concentrations. Segmental effective permeability was derived from Caco-2 apparent permeability (21.3 × 10^−6^ cm/s; propranolol reference). No dose-specific parameters were applied; dose-dependent absorption therefore arises from these fixed inputs, as the administered amount increasingly exceeds the amount that can dissolve within the available intestinal fluid and transit time as dose increases.

Renal clearance was modeled as negligible, consistent with the reported clinical disposition profile. Human urinary recovery was <0.1% of the administered dose, and preclinical rat mass-balance studies showed negligible urinary and biliary excretion, supporting predominantly metabolic clearance. Preclinical metabolism studies identified CYP3A4 as the sole contributor to VU319 metabolism [10], consistent with an estimated fraction metabolized by CYP3A4 approaching unity. 

VU319 is not a substrate of P-glycoprotein (P-gp) or breast cancer resistance protein (BCRP); therefore, these transporters were not included as determinants of VU319 absorption or elimination. The compound has also been reported to inhibit multidrug and toxin extrusion protein 2-K (MATE2-K) and BCRP (IC50 = 0.642 and 1.77 µM, respectively). These findings indicate potential perpetrator activity, although the extent to which transporter interactions contribute to VU319 disposition has not been established. Given the negligible renal excretion and evidence supporting CYP3A4-mediated clearance, the model treats hepatic metabolism as the predominant determinant of systemic clearance, while recognizing that uncharacterized transporter effects may contribute. Tissue distribution was modeled without explicit transporter-mediated uptake or efflux.

Hepatic elimination was modeled using CYP3A4-mediated intrinsic clearance scaled through the standard well-stirred liver model with explicitly specified microsomal unbound fraction (*f*_u,mic_). Renal clearance was assumed negligible, consistent with the reported clinical disposition profile.

### 2.3. Clinical Pharmacokinetic Data

Clinical pharmacokinetic data were obtained from the published Phase 1 single ascending dose (SAD) study of VU319 in healthy volunteers (NCT03220295) [11]. The study was a randomized, double-blind, placebo-controlled trial conducted in 40 healthy participants aged 18–55 years across five dose cohorts (60, 120, 240, 400, and 600 mg; *n* = 6 active and 2 placebo participants per cohort). Participants received a single oral dose under fasted conditions (water permitted) for 8 h before and 4 h after dosing, and each dose was administered orally with water. Plasma samples were collected 0.5 h before dosing and at 0.5, 1, 1.5, 2, 2.5, 3, 5, 7, 9, 12, 24, 26, 28, 36, 48, and 144 h after dosing. The drug formulation was a solid oral dosage form containing micronized drug. In the 600 mg cohort, the 0.5 and 1 h samples were replaced with samples at 72 and 96 h because the half-life was longer than originally estimated [11].

Noncompartmental pharmacokinetic parameters, including C_max_, T_max_, AUC_0–last_, AUC_0–∞_, t_½_, CL/F, and V_z_/F, were reported as arithmetic mean (standard deviation) for each cohort. These parameters were used as reference values for model evaluation. Cohort-specific demographic data, including age, sex distribution, and body weight, were extracted from the published study and used to generate dose-specific virtual populations (Section 2.8). Each virtual population (*n* = 6) was generated in Simcyp using the age range, proportion of females, and body weight range of the corresponding clinical cohort: 60 mg, 23–51 years, 1 of 6 female, 55.8–93.3 kg; 120 mg, 20–51 years, 1 of 6 female, 69.7–95.3 kg; 240 mg, 19–48 years, 1 of 6 female, 74.1–84.6 kg; 400 mg, 21–52 years, 1 of 6 female, 62.7–111.6 kg; and 600 mg, 20–54 years, 2 of 6 female, 50.8–82.6 kg.

### 2.4. In Vitro and Physicochemical Inputs

The baseline PBPK model, prior to any refinement, was defined from experimentally measured physicochemical and in vitro ADME inputs, supplemented by a predicted intrinsic solubility and predicted microsomal unbound fraction (Table 1). Physicochemical input parameters were obtained from the published discovery characterization of VU319 [10] and are summarized in Table 1. Absorption—Absorption was modeled with the ADAM model using the default monodisperse particle radius (10 µm) and a precipitation rate constant of 4 h^−1^. The pH-solubility profile was defined using user-specified values representative of biorelevant media: 0.05 mg/mL at pH 1.6 under FaSSGF conditions, 0.00532 mg/mL at pH 6.5 under FaSSIF conditions, and a predicted intrinsic solubility of 0.00409 mg/mL (consistent with the Henderson–Hasselbalch behavior of a weak base). Permeability was defined using Caco-2 apparent permeability (P_app_ = 21.3 × 10^−6^ cm/s), with propranolol as the reference compound and a permeability scalar of 1. Distribution—Distribution was modeled using the full PBPK model with Method 1 (Poulin & Theil) tissue partitioning with a K_p_ scalar of 1. Distribution was parameterized using the measured plasma unbound fraction (*f*_u_ = 0.034) and a blood-to-plasma ratio (B/P = 1.29). Metabolism—Metabolism was characterized primarily via hepatic metabolism using human liver microsomal intrinsic clearance (32.2 µL/min/mg protein; CYP3A4), corrected for nonspecific microsomal binding using a predicted microsomal unbound fraction (*f*_u,mic_ = 0.41286 at 0.5 mg/mL microsomal protein; Hallifax–Houston method). The CYP3A4 pathway was designated as the primary metabolic route, consistent with evidence indicating CYP3A4 as the predominant pathway for VU319 metabolism. This metabolic activity was scaled through the standard Simcyp IVIVE workflow (using MPPGL, liver weight, and hepatocellularity) via the well-stirred liver model. Excretion—Renal and biliary clearance were assumed to be negligible, based on human urinary recovery of less than 0.1% of the dose. Additionally, no transporter-mediated clearance or distribution processes were included in the baseline model.

### 2.5. IVIVE-Based Reference Predictions

Reference IVIVE-based simulations were generated as the pre-refinement baseline. Tissue distribution was predicted using Method 1 (Poulin & Theil [6]) with no K_p_ scaling applied (K_p_ scalar = 1). Clearance was predicted from human liver microsomal intrinsic clearance (CL_int_ = 32.2 µL/min/mg protein) using the standard Simcyp IVIVE workflow, including MPPGL, liver mass, hepatocellularity scaling, and the well-stirred liver model. Absorption was modeled using the default monodispersed particle radius (10 µm), default precipitation rate constant (PRC = 4 h^−1^), and the experimentally defined pH-solubility profile. PRC and CSR were retained at their Simcyp default values and were not calibrated against the observed PK data. These simulations established the baseline divergence between IVIVE-based predictions and observed pharmacokinetics addressed in subsequent refinement steps.

### 2.6. Sequential Parameter Refinement

Model refinement was performed sequentially, beginning with distribution, followed by clearance, and finally absorption, following the framework described by Santos et al. [3] for PBPK calibration using available clinical data. To limit the risk of overfitting, only three parameters were refined (K_p_ scalar, CL_int_, and particle radius). Each parameter was calibrated against the observed measure it most strongly influences at the 120 mg calibration reference dose: the K_p_ scalar against V_z_/F, CL_int_ against AUC_0–∞_, and particle radius against AUC_0–∞_ and C_max_. Parameters calibrated at earlier steps were held fixed during later steps. Data from the 60, 240, 400, and 600 mg cohorts were not used to estimate any parameter and were reserved for independent verification of the final model (Section 2.8). Each refinement stage evaluated a specific mechanistic hypothesis within a consistent workflow: (i) the initial prediction failed because of the IVIVE assumption or parameter under investigation; (ii) the parameter was modified based on mechanistic rationale and literature support; (iii) the modification produced a quantitative change in agreement between predicted and observed pharmacokinetics; and (iv) the resulting improvement informed mechanistic interpretation of the observed divergence. Distribution, clearance, and absorption parameters were estimated sequentially and independently rather than simultaneously, to limit the extent to which any one parameter can compensate for error in another.

The 120 mg fasted cohort was used as the calibration reference throughout the study because it lies within the lower region of the SAD range, where absorption is expected to be less constrained by solubility than at higher doses. Additionally, 120 mg had the second-lowest dose number in the SAD range (Do = 90 based on FaSSIF solubility), half that at 240 mg and less than one-third of those at 400 and 600 mg (Do = 301 and 451), indicating a smaller excess of administered drug relative to dissolvable drug than at the higher doses. This dose was therefore used to first calibrate distribution using the observed V_z_/F and clearance using AUC_0–∞_, before adjusting absorption parameters. This approach allowed distribution and clearance to be characterized before accounting for dissolution-limited absorption. Calibration at 120 mg also provided an independent test of model performance across the remaining dose range, with AUC_0–∞_ predictions remaining within 6% of observed values through 240 mg.

Although calibration at 400 or 600 mg could improve numerical fit at the higher doses, these cohorts are expected to be more strongly solubility-limited. Using either as the calibration dose could therefore incorporate high-dose absorption limitations into the distribution and clearance adjustments, making it more difficult to distinguish the contribution of each process. The 120 mg reference was therefore selected to allow distribution and clearance to be calibrated before accounting for high-dose, dissolution-limited absorption, with the higher-dose cohorts reserved for independent evaluation of model performance.

#### 2.6.1. Distribution Refinement—K_p_ Scalar

The first hypothesis (H1) was that V_ss_ prediction by Method 1 does not fully capture the observed disposition, given the compound’s lipophilicity, fraction unbound (*f*_u_), and ionization profile.

Poulin & Theil was selected because VU319 is highly lipophilic (logP = 4.18) and minimally ionized at physiological pH (pK_a_ 1.4), conditions in which Rodgers & Rowland may overpredict tissue partitioning. Residual V_ss_ overprediction was corrected by empirical calibration of the K_p_ scalar [6].

The K_p_ scalar was empirically calibrated from 0.05 to 0.25 in 0.025 increments against the observed V_z_/F at 120 mg (107 L), while all other inputs remained fixed at IVIVE reference values. The observed V_z_/F at 120 mg was used as the upper boundary for model V_ss_, since model V_ss_ > V_z_/F would imply F > 1, which is physiologically unlikely. The scalar producing physiologically plausible V_ss_ and terminal half-life estimates relative to observed data was carried forward into subsequent analyses. Adjustment of the K_p_ scalar should therefore be interpreted as an empirical correction to method 1 partitioning rather than a mechanistically derived parameter.

#### 2.6.2. Clearance Refinement—Microsomal Cl_int_ Correction

The second hypothesis (H2) was that standard IVIVE scaling of microsomal CL_int_ overpredicts in vivo hepatic clearance for VU319.

The required downward CL_int_ adjustment was interpreted within the reported HLM/HH disconnect framework for lipophilic substrates [3]. With the K_p_ scalar fixed from Stage 1, CL_int_ was evaluated from 2 to 10 µL/min/mg protein in 1 µL/min/mg increments while retaining the predicted *f*_u,mic_ value of 0.41286. The CL_int_ value producing an AUC_0–∞_ closest to the observed 120 mg exposure (59.0 µg·h/mL) was selected. Selection emphasized mechanistic plausibility rather than minimization of AUC residual error.

#### 2.6.3. Absorption Refinement (Particle Radius)

The third hypothesis (H3) was that default absorption parameters do not adequately capture formulation dissolution kinetics, resulting in poor recovery of observed exposure and dose proportionality.

With K_p_ scalar and CL_int_ fixed at calibrated values, monodispersed particle radius was evaluated at 1, 3, 5, 10, 25, 50, and 100 µm while all other ADAM inputs remained at default settings (PRC = 4 h^−1^, CSR = 10, bile micelle solubilization predicted). The particle radius producing the closest simultaneous agreement with observed AUC_0–∞_ and C_max_ at the 120 mg calibration dose was selected; T_max_ was also considered. The selected radius represents a model assumption, as the formulation particle size distribution was not available.

### 2.7. Sensitivity Analysis

Local sensitivity for each refined parameter was assessed using the calibration analyses described in Section 2.6.1, Section 2.6.2 and Section 2.6.3. Sensitivity to AUC and C_max_ was quantified as the range of model response across the mechanistically plausible values tested for each parameter. Parameters producing larger changes in model output were considered more sensitive.

A targeted post-calibration sensitivity analysis was also performed on the ADAM critical supersaturation ratio (CSR) to assess whether prolonged supersaturation could explain the residual underprediction at higher dose ranges. In the Simcyp ADAM model, the supersaturation ratio (SR) is the dissolved drug concentration in each gut segment divided by the equilibrium solubility under the local pH and bile salt conditions. Precipitation begins only when SR exceeds the CSR, after which dissolved drug precipitates at a first-order rate governed by the precipitation rate constant (PRC). CSR was set to 10 (Simcyp default), 25, 50, and 100 in the cohort-matched 600 mg population (*n* = 6), with all other inputs held at calibrated values (particle radius 1 µm; PRC 4 h^−1^; measured pH-solubility profile; bile micelle solubilization predicted from logP; fluid intake 250 mL).

### 2.8. Model Verification Using Independent Dose Cohorts

All simulations used the Simcyp Healthy Volunteers population, with the same random-sampling settings applied throughout. Model refinement was performed at the 120 mg dose using the population-representative individual (one trial of one subject; male, 23.5 years, 80.7 kg), which carries no inter-individual variability. The final model was then verified using separate cohort-matched virtual populations, each comprising one trial of six subjects and generated using the age range, proportion of females, and body weight range of the corresponding clinical cohort (Section 2.3). The populations used for refinement and for verification were therefore different. In the cohort-matched populations, default Simcyp inter-individual variability was retained for physiological parameters, enzyme abundances, fluid intake with dose (CV 30%), gastric residence time (CV 36%), small-intestinal residence time (CV 9.2%), and micelle diffusion coefficient (CV 20%); no variability was applied to CL_int_ or effective permeability.

Predicted geometric mean AUC_0–∞_ and arithmetic mean C_max_ were compared with the observed values reported for each cohort [11]. Model performance was assessed using the geometric mean fold error (GMFE) and the percentage of doses predicted within 2-fold of observed values, with a GMFE below 2 and prediction within 2-fold predefined as performance thresholds. Performance metrics were calculated for the four verification doses (60, 240, 400, and 600 mg), which were not used during refinement. The 120 mg calibration dose is shown for completeness but was excluded from the verification metrics.

Performance was assessed using geometric mean fold error (GMFE) and the percentage of doses predicted within 2-fold of observed values; consistent with FDA PBPK guidance on the reporting of PBPK analyses, the clinical data used for parameter optimization are identified, and model performance was evaluated against clinical data not used during refinement [12]. Simulated profiles (mean and 5th–95th percentile interval) were compared with observed mean ± SD concentrations, and a visual predictive check was constructed by overlaying the simulated interval on observed individual concentrations and the observed median at each nominal sampling time (Section 3.6). The dose number (Do) was calculated as Do = (M_0_/V_0_)/C_s_, where M_0_ is the administered dose (mg), V_0_ is the fluid volume taken with the dose (250 mL, as in the Simcyp simulations), and C_s_ is the solubility (mg/mL) [13]. FaSSIF solubility was used as the primary input because the small intestine is the main site of absorption; FaSSGF values are shown for comparison.

## 3. Results

### 3.1. IVIVE-Based Reference Predictions and Observed Divergence

Pre-refinement IVIVE predictions diverged substantially from observed pharmacokinetics across all three ADME domains at the 120 mg calibration dose. Method 1 predicted V_ss_ = 7.895 L/kg (552 L for a 70 kg reference adult), greatly exceeding the observed V_z_/F of 107 L and implying implausible bioavailability, since model V_ss_ > V_z_/F requires F > 1.

HLM-derived hepatic clearance predicted CL = 9.71 L/h, approximately 4.7-fold higher than the observed CL/F of 2.07 L/h assuming F = 1, with larger discrepancies at physiologically plausible F values. The model also underpredicted AUC_0–∞_ at 120 mg, yielding 5.03 µg·h/mL versus the observed 59.0 µg·h/mL, corresponding to an 11.7-fold error.

The consistent direction of these discrepancies supported sequential refinement of model parameters rather than modification of the PBPK model structure.

### 3.2. Distribution Refinement

**Figure 2:**
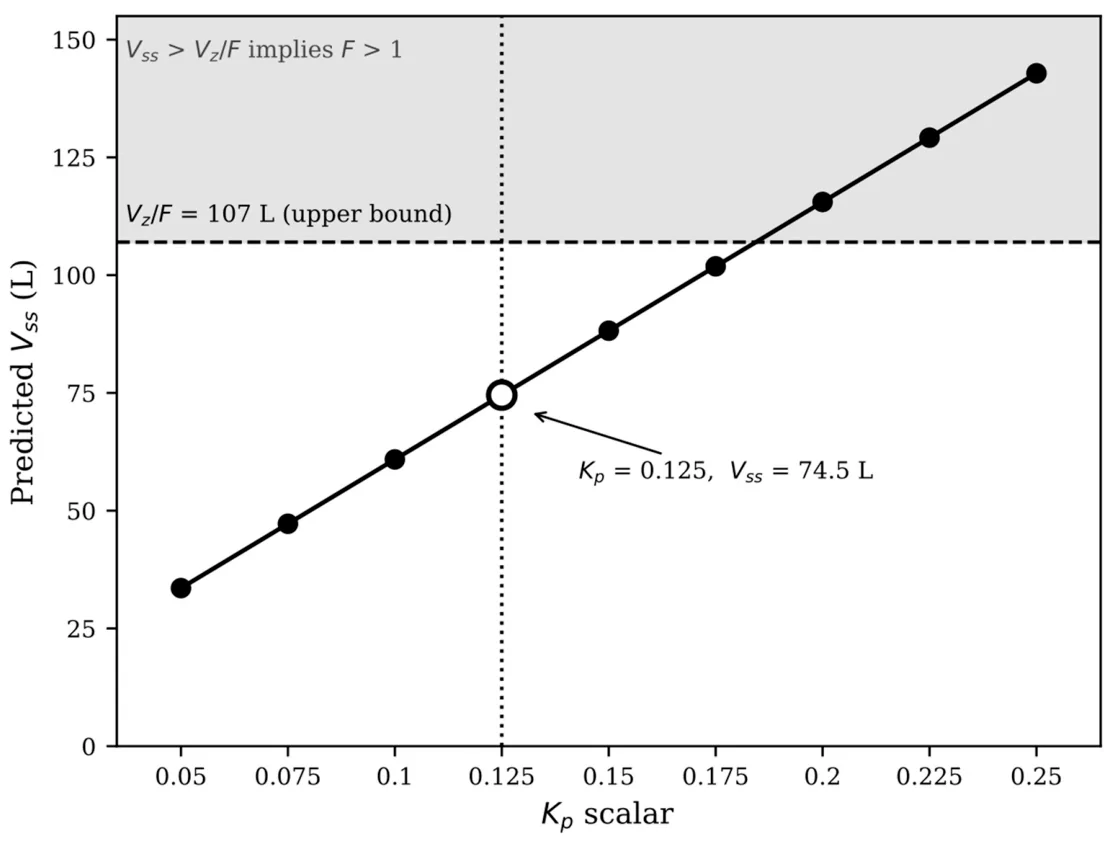
Effect of global K_p_ scalar on predicted V_ss_ (L) for VU319 during distribution refinement (120 mg oral, single dose, fasted; Simcyp V24 population-representative simulation). Filled circles denote predicted V_ss_ values at each tested K_p_ scalar, connected by the solid line. The dashed line indicates the observed V_z_/F (107 L; 120 mg SAD cohort, mean, *n* = 5) [11], which serves as a physiological upper bound: because V_z_ ≥ V_ss_ by definition, values above this threshold imply F > 1 and are physiologically excluded. The dotted vertical line marks the calibrated K_p_ scalar (0.125). The gray shaded region denotes the physiologically excluded range where V_ss_ exceeds observed V_z_/F; the white region denotes the physiologically acceptable range. The open circle denotes the selected calibration value (K_p_ = 0.125, V_ss_ = 74.5 L), retained for subsequent clearance refinement. K_p_, tissue-to-plasma partition coefficient; V_ss_, volume of distribution at steady state; V_z_/F, apparent volume of distribution during the terminal phase; SAD, single ascending dose.

K_p_ scalar calibration produced linear changes in model V_ss_ across the tested range (Figure 2). The observed V_z_/F of 107 L defined the upper physiologic limit, excluding K_p_ scalar values ≥0.20 because they produced V_ss_ values >107 L, which would imply F > 1.

Within the acceptable range, a K_p_ scalar of 0.125 was selected, yielding a model V_ss_ of 1.065 L/kg (74.5 L for a 70 kg reference adult). This corresponds to an 8-fold reduction from the Method 1 baseline and is consistent with empirical corrections reported for highly lipophilic, low-*f*_u_ compounds. AUC_0–∞_ remained unchanged across K_p_ scalar values, confirming that K_p_ refinement affected distribution without altering clearance.

Taken together, these results partially supported H1, identifying distribution as a secondary contributor. The K_p_ scalar required to recover observed V_ss_ (×0.125) is consistent with the recognized tendency of tissue composition-based methods to overpredict tissue partitioning for highly lipophilic, minimally ionized compounds [6,7], and the refined V_ss_ (74.5 L) falls within a physiologically plausible range for a compound of this physicochemical class. Distribution adjustment improved overall model alignment but did not resolve the IVIVE-observed divergence, indicating that additional mechanistic contributors require investigation.

### 3.3. Clearance Refinement

**Figure 3:**
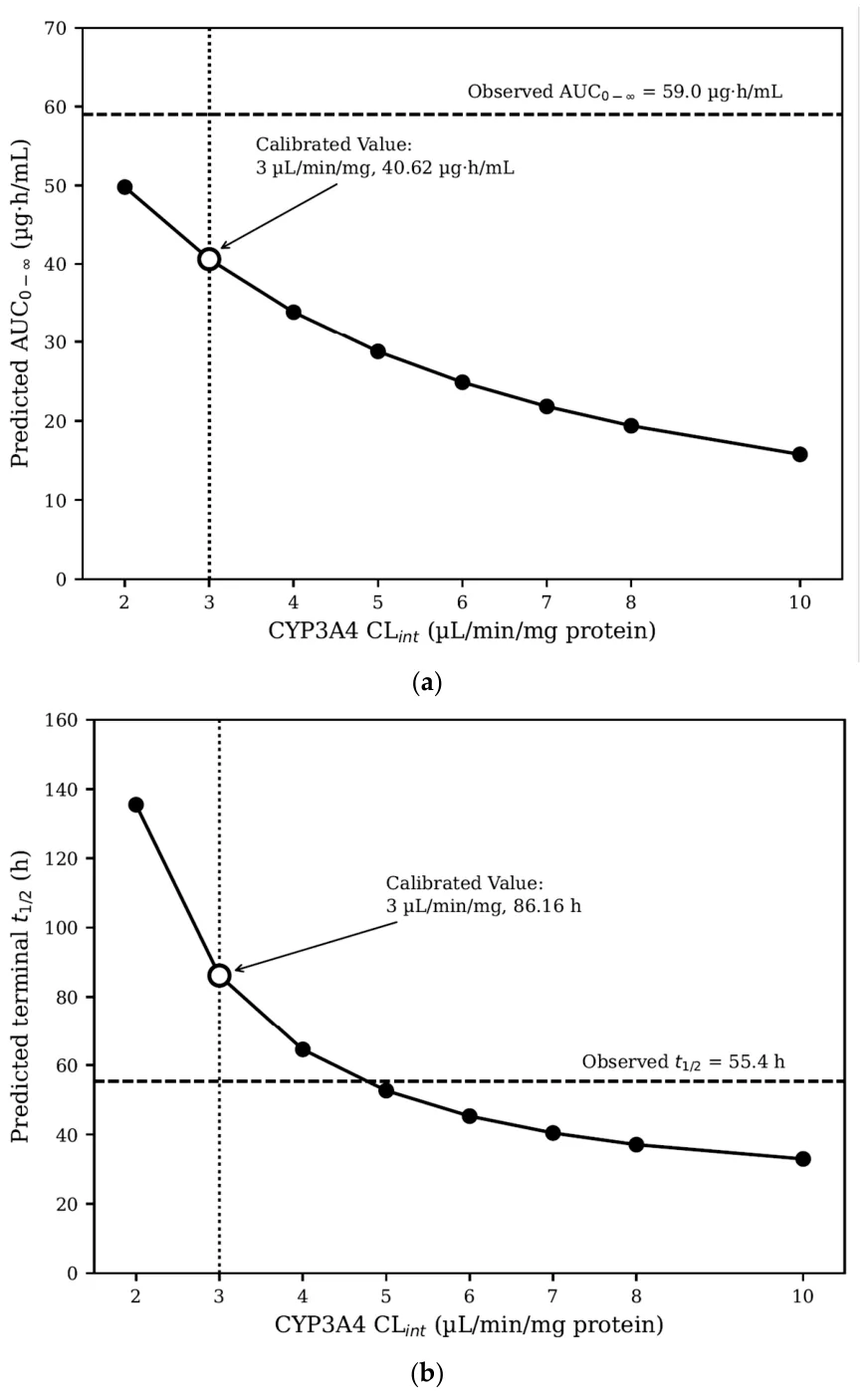
(**a**) Effect of CYP3A4 CL_int_ on predicted AUC_0–∞_ (µg·h/mL) for VU319 (120 mg oral, single dose, fasted; Simcyp V24 population-representative simulation with refined K_p_ scalar = 0.125). Filled circles denote predicted AUC_0–∞_ values at each tested CL_int_, connected by the solid line, and the open circle denotes the calibrated value. The dashed horizontal line denotes the observed AUC_0–∞_ (59.0 µg·h/mL; 120 mg SAD cohort, mean, *n* = 5) [11]. The dotted vertical line marks the calibrated CL_int_ (3 µL/min/mg protein), selected to optimize systemic exposure recovery prior to absorption refinement. Predicted AUC_0–∞_ decreases monotonically with increasing CL_int_, consistent with metabolism-driven elimination. (**b**) Effect of CYP3A4 CL_int_ on predicted terminal half-life (h) for VU319 (120 mg oral, single dose, fasted; Simcyp V24 population-representative simulation with refined K_p_ scalar = 0.125). Filled circles denote predicted t½ values at each tested CL_int_, connected by the solid line, and the open circle denotes the calibrated value. The dashed horizontal line denotes the observed t½ (55.4 h; 120 mg SAD cohort, mean, *n* = 6, SD = 46.8) [11]; the wide variability reflects inter-subject differences in the terminal phase. The dotted vertical line marks the calibrated CL_int_ (3 µL/min/mg protein). At the calibrated value, the plotted t½ (86.16 h) exceeds the observed mean; plotted values were estimated from the terminal slope of the sampled simulated profile, which at the default 10 µm particle radius also reflects continued absorption. The terminal elimination half-life at the calibrated CL_int_ was 62.3 h and was unchanged by subsequent particle-radius refinement (Section 3.5).

Calibration of CL_int_ produced a monotonic decrease in model AUC_0–∞_ as CL_int_ increased, with the observed 120 mg AUC_0–∞_ (59.0 µg·h/mL) falling between CL_int_ values of 2 µL/min/mg (AUC 80.98 µg·h/mL) and 3 µL/min/mg (AUC 53.99 µg·h/mL) (Figure 3a). A CL_int_ value of 3 µL/min/mg was selected, corresponding to a 10.7-fold reduction from the in vitro HLM estimate. The calibrated model implied a bioavailability of approximately 0.44. Increasing CL_int_ also shortened the predicted terminal half-life (Figure 3b), consistent with its expected effect on systemic elimination.

These results supported H2, and the mechanistic interpretation of the correction magnitude is discussed in Section 4.2.

### 3.4. Absorption Refinement

**Figure 4:**
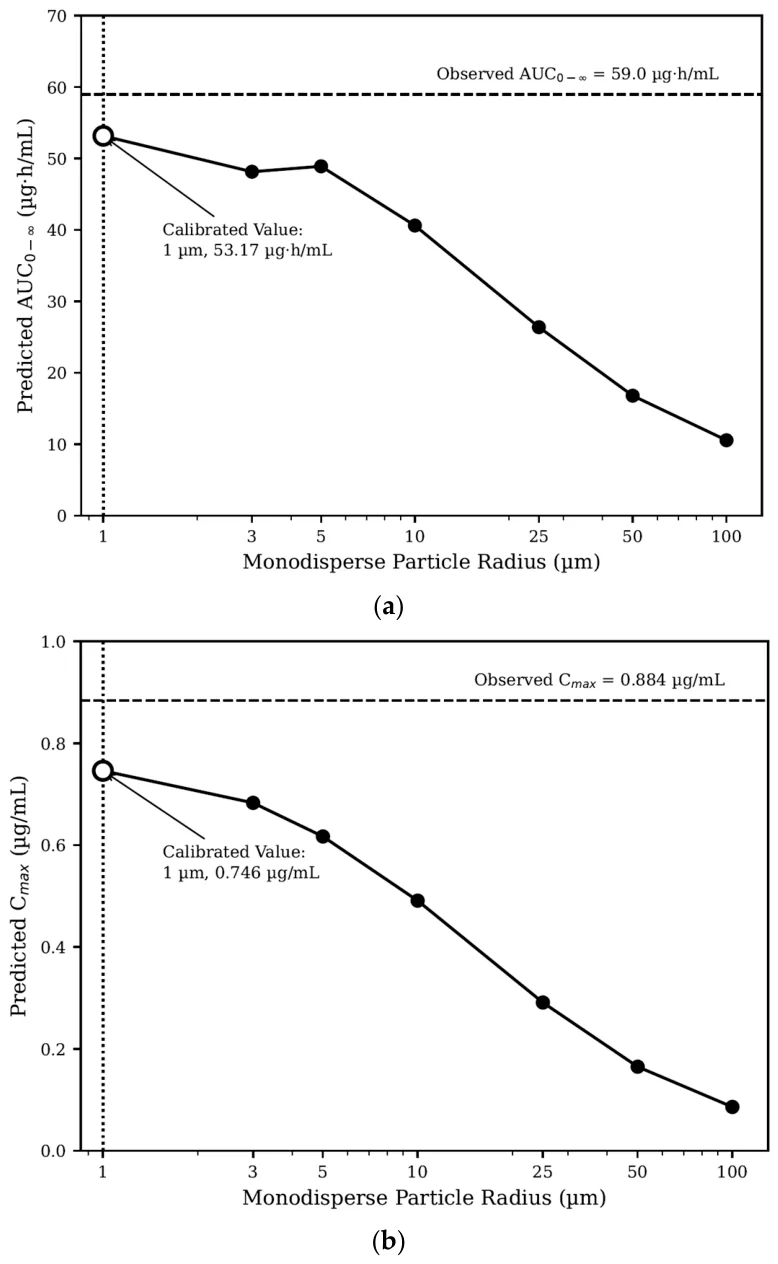
(**a**) Effect of monodispersed particle radius on predicted AUC_0–∞_ (µg·h/mL) for VU319 (120 mg oral, single dose, fasted; Simcyp V24 population-representative simulation with refined K_p_ scalar = 0.125 and CYP3A4 CL_int_ = 3 µL/min/mg protein). Filled circles denote predicted AUC_0–∞_ values at each tested monodispersed particle radius, connected by the solid line, and the open circle denotes the calibrated value. The dashed horizontal line denotes the observed AUC_0–∞_ (59.0 µg·h/mL; 120 mg SAD cohort, mean, *n* = 5) [11]. The dotted vertical line marks the calibrated particle radius (1 µm), which produced the closest agreement with both observed AUC_0–∞_ and observed C_max_ across the evaluated range. Predicted AUC_0–∞_ decreases monotonically with increasing particle radius, consistent with progressively rate-limited dissolution at larger particle sizes. (**b**) Effect of monodispersed particle radius on predicted C_max_ (µg/mL) for VU319 (120 mg oral, single dose, fasted; Simcyp V24 population-representative simulation with refined K_p_ scalar = 0.125 and CYP3A4 CL_int_ = 3 µL/min/mg protein). Filled circles denote predicted C_max_ values at each tested monodispersed particle radius, connected by the solid line, and the open circle denotes the calibrated value. The dashed horizontal line denotes the observed C_max_ (0.884 µg/mL; 120 mg SAD cohort, mean, *n* = 6) [11]. The dotted vertical line marks the calibrated particle radius (1 µm), selected jointly with Figure 4a as the value producing the closest agreement with both observed exposure metrics. Predicted C_max_ decreases monotonically with increasing particle radius, mirroring the AUC_0–∞_ response and reflecting the shared sensitivity of both metrics to in vivo dissolution rate.

With distribution and clearance fixed at calibrated values, particle radius optimization identified that 1 µm provided the best overall agreement with observed AUC_0–∞_ and C_max_ at 120 mg. This refinement from the Simcyp default value of 10 µm is consistent with the use of micronized active pharmaceutical ingredient (API) formulations in BCS Class II drug development [4]. Predicted AUC_0–∞_ and C_max_ both decreased with increasing particle radius (Figure 4a,b), demonstrating the sensitivity of exposure to dissolution rate.

Overall, these results supported H3, identifying dissolution-limited absorption as a major contributor to the IVIVE–observed divergence at the calibration dose.

### 3.5. Sensitivity Assessment via Parameter Calibration

Local sensitivity for each refined parameter was quantified as the range of model response across the tested calibration values at 120 mg (Table 2). Because the tested ranges differ between parameters, these values indicate the relative influence of each parameter rather than a strict ranking. CL_int_ had the largest effect on AUC_0–∞_ (approximately 5-fold across 2–10 µL/min/mg) but little effect on C_max_ (1.1-fold). Particle radius had the largest effect on C_max_ (approximately 9-fold across 1–100 µm) and also altered AUC_0–∞_ approximately 4-fold. K_p_ scalar had no direct effect on AUC_0–∞_ and primarily influenced V_ss_ and terminal half-life. This lack of an effect on AUC indicates that distribution and clearance were separately identifiable, such that adjusting one did not compensate for error in the other.

CL_int_ and particle radius both influenced AUC_0–∞_ but could be distinguished by their effects on C_max_ and half-life. Particle radius had a much larger effect on C_max_ than CL_int_. In contrast, CL_int_ changed half-life 4.8-fold, whereas terminal elimination half-life was unchanged between particle radii of 1 and 10 µm (62.3 h). As a result, a different combination of CL_int_ and particle radius that matched the observed AUC_0–∞_ would be unlikely to also match the observed C_max_ and half-life. Consistent with this, the calibrated model reproduced AUC_0–∞_ within 6% at 60 and 240 mg, doses that were not used during refinement.

Fold-change is the ratio of the larger to the smaller model output across the evaluated input range (maximum ÷ minimum) and quantifies parameter sensitivity; it is distinct from the model-versus-observed fold error reported in Section 3.6. AUC_0–∞_ and C_max_ both decrease as each parameter increases; for the secondary outputs, the direction of change is indicated as “higher” or “lower” with increasing parameter value.

### 3.6. Model Verification Across Independent SADs

**Figure 5:**
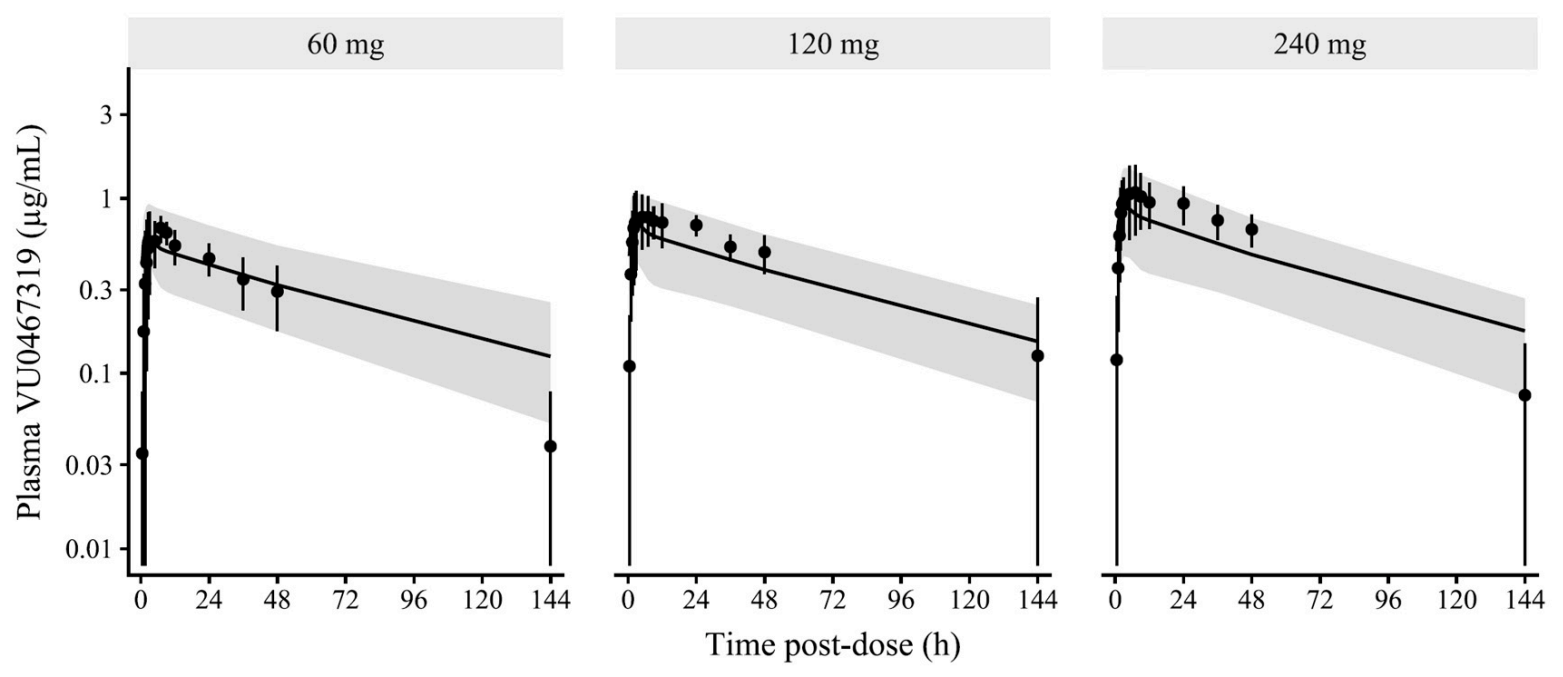
Simulated versus observed plasma VU319 concentration–time profiles following single oral doses of 60, 120, and 240 mg under fasted conditions. Solid black lines represent the simulated geometric mean plasma concentration from cohort-matched virtual populations (*n* = 6 per dose) generated using the calibrated PBPK model (Simcyp V24; K_p_ scalar = 0.125, CYP3A4 CL_int_ = 3 µL/min/mg protein, monodispersed particle radius = 1 µm). Gray shading represents the simulated 5th–95th percentile band. Filled circles and error bars represent the observed arithmetic mean ± SD from the VU319 single ascending dose (SAD) study (NCT03220295; *n* = 6 per cohort, fasted). At the 240 mg/144 h time point, observed values are based on *n* = 4; two subjects had plasma concentrations below the lower limit of quantification. Plasma concentration is displayed on a log10 scale.

The calibrated model was verified against observed pharmacokinetics across all five SAD cohorts using cohort-matched virtual populations (*n* = 6 per dose) (Table 3, Figure 5). Geometric mean AUC_0–∞_ predictions were within 6% of observed values for the 60, 120, and 240 mg cohorts, indicating that calibration at 120 mg generalized well across the lower dose range. The observed clinical profile showed sub-proportional increases in exposure with increasing dose, whereas the refined model progressively underpredicted exposure at higher doses.

The 400 and 600 mg concentration-time profiles, shown in Supplementary Figure S1, showed similar dose-dependent divergence, with more sustained observed profile at higher doses. At 400 mg, the model underpredicted AUC_0–∞_ and C_max_ by 1.57-fold and 1.72-fold, respectively, both within the 2-fold acceptance criterion. At 600 mg, AUC_0–∞_ and C_max_ were underpredicted by 2.44-fold and 2.50-fold, respectively, slightly exceeding the 2-fold threshold. Thus, model performance remained within the predefined criterion through 400 mg but deteriorated at 600 mg. The simulated concentration-time profiles showed a faster onset of exposure than was observed clinically, with simulated concentrations higher than observed during the first 1 to 2 h but lower from approximately 12 to 48 h. At 48 h, the simulated/observed concentration ratio decreased progressively with dose, from approximately 1.1 at 60 mg to 0.28 at 600 mg, while the 600 mg observed profile remained relatively constant from 2.5 to 48 h. Despite these profile differences, overall AUC at 60–240 mg remained relatively close to observed values, indicating that agreement in AUC does not necessarily reflect accurate prediction of the timing and duration of drug exposure.

Across the four verification doses (60, 240, 400, and 600 mg), GMFE was 1.45 for AUC_0–∞_ and 1.56 for C_max_, and three of four doses (75%) were predicted within 2-fold for both parameters. Including the 120 mg calibration dose, GMFE across all five doses was 1.36 for AUC_0–∞_ and 1.46 for C_max_, with four of five doses (80%) predicted within 2-fold for both parameters. Overall, the model met predefined performance thresholds while showing progressive underprediction at the higher doses (≥400 mg). This agreement indicates that the model reproduces systemic exposure across this dose range, but does not by itself establish the proposed mechanism.

**Figure 6:**
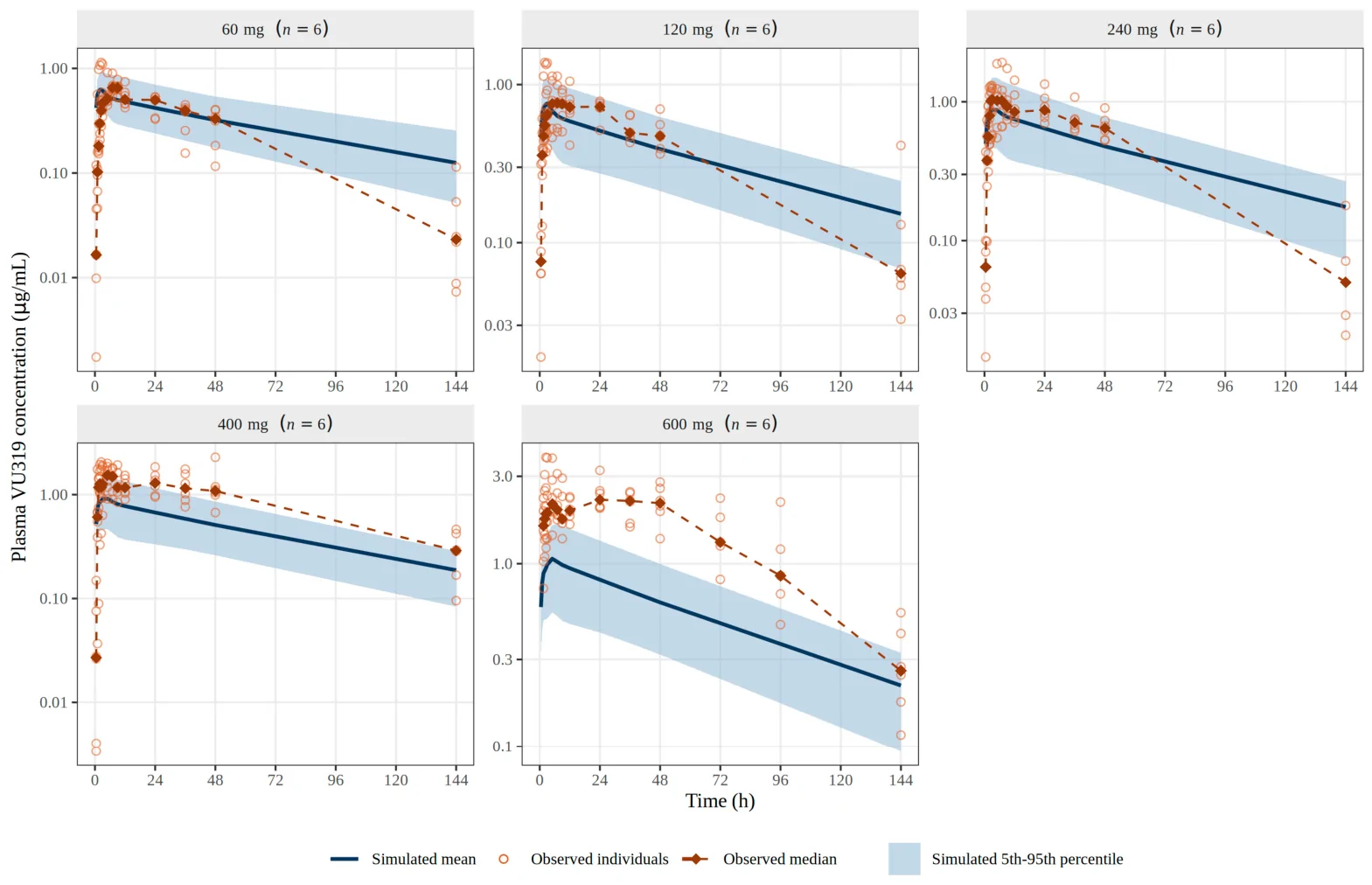
Visual predictive check of the calibrated VU319 PBPK model against observed single ascending dose (SAD) pharmacokinetics for each fasted dose cohort. Plasma VU319 concentrations (µg/mL) are shown on a semi-logarithmic scale versus time. The solid line denotes the simulated population mean and the shaded region the simulated 5th–95th percentile prediction interval, generated from cohort-matched virtual populations (Simcyp Simulator V24; one trial of six subjects per dose, with age range, sex proportion and body weight matched to the corresponding clinical cohort). Open circles show individual observed concentrations; filled diamonds, connected by a dashed line, show the observed median at each nominal sampling time. Observed data are from the Phase 1 SAD study (NCT03220295). The 120 mg cohort served as the calibration reference, and the remaining cohorts represent independent model evaluation. Terminal-phase sampling was sparse (a single 144 h sample per participant, two of which at 240 mg were below the lower limit of quantification; 72 and 96 h sampled only in the 600 mg cohort) and terminal half-life was highly variable across participants; terminal-phase comparisons should therefore be interpreted with caution.

The visual predictive check (Figure 6) showed that, after the absorption phase (3–48 h), 85% of observed concentrations at 60, 120, and 240 mg fell within the simulated 5th–95th percentile interval, and the observed median remained within the interval at every sampling time. Over the same period, 54% of observations at 400 mg and 94% at 600 mg exceeded the upper bound, with the 600 mg observed median above the interval at every time from 1.5 to 48 h, consistent with the progressive underprediction described above. At 144 h, 11 of 16 quantifiable observed concentrations at 60–240 mg fell below the simulated interval, indicating that the model overpredicts late terminal-phase concentrations, consistent with simulated terminal half-lives that exceeded the observed cohort means at every dose (median simulated values 55–69 h versus observed means of 30.4–55.4 h).

To investigate the mechanism underlying the high-dose underprediction, CSR was varied from the default value of 10 to 25, 50, and 100 in the cohort-matched 600 mg population (*n* = 6), with all other inputs held at calibrated values. Increasing CSR from 10 to 25 increased the geometric mean AUC_0–∞_ from 89.66 to 90.22 µg·h/mL (0.6%), the arithmetic mean C_max_ from 1.127 to 1.138 µg/mL (0.9%), and the mean fraction absorbed from 0.150 to 0.151. Further increases in CSR to 50 and 100 produced no additional change in any of these outputs. Only two of six subjects were affected, with individual AUC_0–∞_ increases of 0.4% and 3.4%. The absence of any change above CSR 25 indicates that predicted SR did not exceed 25 in any subject, and exceeded 10 in only two. Within the model, supersaturation therefore does not appear to be a dominant driver of the high-dose underprediction.

Dose numbers and dose-normalized exposure across the SAD range are summarized in Table 4. Dose numbers exceeded 1 at every dose using either FaSSIF or FaSSGF solubility. Observed dose-normalized AUC_0–∞_ decreased by approximately half between 60 and 240 mg and changed marginally between 240 and 600 mg.

## 4. Discussion

### 4.1. Dissolution-Limited Absorption as the Primary Supported Contributor

Although the essentially unionized state of VU319 favors membrane permeation, its high measured permeability indicates that permeability is not rate-limiting for absorption. Instead, its unionized, lipophilic character contributes to low aqueous solubility, making dissolution rather than ionization or permeability the primary supported absorption limitation and a determinant of bioavailability.

Particle radius refinement produced a substantial model response across the tested calibration ranges and yielded the greatest improvement in observed–predicted agreement at the calibration dose. Particle size is a recognized formulation attribute that can materially influence dissolution and oral absorption in mechanistic physiologically based biopharmaceutics modeling (PBBM) of immediate-release products, for which particle-size distribution may be incorporated as a model input [14]. In the present study, however, the formulation particle-size distribution was not derived from experimental data; the calibrated 1 µm radius should therefore be interpreted as part of a calibrated mechanistic hypothesis rather than a measured formulation attribute. Because compound-specific precipitation data were unavailable, PRC was retained at the Simcyp default value rather than calibrated to clinical PK data, consistent with evidence that precipitation is not universal among weakly basic, water-insoluble IR compounds and that PRC should be informed by experimental evidence [15].

The simulated concentration–time profiles reached T_max_ earlier than the observed median values (3.2 h at the calibrated 1 µm radius versus 5.0–9.5 h), although observed T_max_ varied widely across cohorts (2–48.2 h), encompassing the simulated value. This difference indicates that the model did not fully reproduce the observed absorption profile but does not appear to be explained by particle radius, as predicted T_max_ was relatively insensitive to particle radius (3.1–4.2 h) and showed no monotonic relationship across the tested range (Supplementary Figure S2).

Within the model, varying CSR from 10 to 100 changed predicted exposure at 600 mg by less than 1%, suggesting that sustained supersaturation did not contribute materially to the high-dose underprediction under the assumed solubility and precipitation inputs. The limited supersaturation is consistent with the ratio of gastric to intestinal solubility (0.05/0.00532 ≈ 9.4), which provides an approximate upper bound on the supersaturation generated when dissolved drug empties from the stomach. Supersaturation is not expected to be greater at lower doses, because every dose studied exceeds the amount that can dissolve in gastric fluid (approximately 12.5 mg in 250 mL). In the small intestine, a FaSSIF solubility of 0.00532 mg/mL and an estimated volume of 105 mL limit dissolved drug to approximately 0.56 mg at any time, less than 0.1% of a 600 mg dose. These findings support solubility-limited absorption as an important contributor to exposure across the dose range, although the greater-than-predicted exposure above 240 mg suggests that additional processes also contribute at the highest doses.

The dose number exceeded 45 at every dose studied (Table 4), consistent with solubility-limited absorption across the full dose range. Under these conditions, the amount absorbed is expected to become largely independent of dose [13], which agrees with the approximately 50% decrease in dose-normalized AUC_0–∞_ between 60 and 240 mg. Above 240 mg, exposure was approximately dose-proportional [11], suggesting that additional processes contribute at higher doses.

Several mechanisms may contribute to the underprediction at 400 and 600 mg. Because the model predicts near-complete gut and hepatic bioavailability (F_g_ ≈ 1.00; F_h_ ≈ 0.99), the underprediction may reflect either underpredicted absorption or reduced systemic clearance. Terminal half-life did not increase at the highest dose (30.4 h at 600 mg versus 31.5–55.4 h at lower doses) [11], which does not suggest substantial saturation of CYP3A4-mediated metabolism, although a contribution cannot be excluded.

Several observations are more consistent with underpredicted absorption. Assuming dose-independent clearance, the fraction absorbed required to reproduce observed exposure remained relatively constant between 240 and 600 mg (approximately 0.30–0.37), whereas the model predicted a decline from 0.32 to 0.15. In addition, observed concentrations at 600 mg were sustained from approximately 2.5 to 48 h, the period in which the model underpredicted most. Together, these findings suggest that absorption may have continued for longer at high doses than the model assumes. Possible explanations include slower gastric emptying or longer intestinal transit of the larger solid dose, and greater in vivo solubilization by bile micelles, as these inputs were held at fasted-state defaults that did not vary with dose.

Other mechanisms appear less likely. Within the model, varying the critical supersaturation ratio changed predicted exposure by less than 1% (Section 3.6), suggesting that precipitation had a limited influence. Permeability is also unlikely to explain a dose-dependent difference, as VU319 is highly permeable and is not a substrate of P-gp or BCRP. Formulation-related effects could not be evaluated because the formulation composition and particle size distribution were not available. The refined model is therefore best regarded as an exposure-scaling and investigative tool rather than a profile-level predictive model.

### 4.2. Clearance Contribution: HLM/HH Disconnect Interpretation

The 10.7-fold downward correction indicates that HLM overpredicted in vivo clearance for VU319. This direction is atypical, as microsomal IVIVE underpredicts in vivo CL_int_ on average, and no association between prediction bias and plasma *f*_u_ has been identified [16]. Overprediction from HLM has instead been attributed to an HLM/HH disconnect arising from permeability-limited hepatocyte uptake and cofactor-limited metabolism [3].

This interpretation is supported by preclinical data, in which in vivo unbound CL_int_ in rat, dog, and cynomolgus monkey was approximately 0.6-fold of the corresponding microsomal value [10]. Additional contributors were evaluated. Because *f*_u,mic_ was predicted (0.41), error in the microsomal binding correction can account for no more than 2.4-fold of the discrepancy. As VU319 is a low-extraction compound, hepatic clearance is governed by the product *f*_u,b_ · CL_int_, and error in the measured plasma *f*_u_ (0.034) therefore cannot be distinguished from error in CL_int_. Variability in MPPGL, liver weight, and CYP3A4 abundance typically contributes less than twofold uncertainty. VU319 is not a substrate of P-gp or BCRP, and its hepatic uptake transporter status has not been characterized.

The magnitude of the correction may also partly reflect bioavailability, because CL_int_ was calibrated against oral AUC_0–∞_ before absorption refinement. However, the terminal elimination half-life, which is independent of F, was 62.3 h at the calibrated CL_int_ (Section 3.5), close to the observed mean of 55.4 h (SD 46.8 h), indicating that the 10.7-fold correction largely reflects clearance rather than bioavailability. The calibrated CL_int_ is therefore best regarded as an empirical value that compensates for uncertainty in binding, scaling factors, and bioavailability, rather than a corrected estimate of microsomal activity. Measurement of *f*_u,mic_ and hepatocyte CL_int_, together with intravenous data to separate clearance from bioavailability, as obtained in combined oral and intravenous studies [17], would likely allow these contributions to be resolved.

### 4.3. Distribution Contribution: Modest but Mechanistically Coherent

The selected K_p_ scalar of 0.125 represents an 8-fold reduction from the Method 1 baseline and is consistent with known overprediction by mechanistic K_p_ methods for highly lipophilic compounds. This correction reflects inherent limitations of tissue composition-based prediction methods rather than inappropriate method selection.

Distribution refinement was necessary to satisfy the physical constraint imposed by observed V_z_/F and to recover the terminal disposition phase, but it was not the primary driver of IVIVE–observed agreement. AUC_0–∞_ remained independent of K_p_ scalar, confirming that distribution primarily affected V_ss_ and terminal half-life rather than overall exposure.

### 4.4. PBPK as an Investigative Tool

The sequential refinement strategy helped distinguish the contributions of distribution, clearance, and absorption to the IVIVE–observed discrepancy. Distribution was refined using V_z_/F, clearance using AUC_0–∞_, and absorption using AUC_0–∞_, C_max_, and T_max_. Sensitivity analysis supported this refinement hierarchy. The K_p_ correction reduced the overprediction of distribution, while the CL_int_ correction reduced the overprediction of in vivo clearance from microsomal IVIVE. Particle radius refinement produced the greatest response among the evaluated absorption parameters at the calibrated dose, supporting a substantial contribution of dissolution kinetics to exposure. At higher doses, the residual underprediction may reflect absorption that continued for longer than the model assumes, although other contributors cannot be excluded (Section 4.1). The CSR analysis further demonstrates how competing mechanistic hypotheses can be evaluated within a unified PBPK framework. These findings support the use of PBPK as an investigative framework for formulation optimization and evaluation of repeated-dose scenarios, although predictive performance under multiple ascending-dose conditions remains to be established.

Divergence between in vitro or computational predictions and in vivo behavior is not limited to PBPK modeling. Differences observed in microsomal systems, including sex-dependent differences in CL_int_, may not be reflected in in vivo pharmacokinetics [17], and computational screening based on predicted oral bioavailability may not reflect the in vivo activity of compounds without confirmation against constituents experimentally detected in blood [18].

### 4.5. Translational Implications

This workflow may apply to other low-solubility compounds through interpretation of divergence patterns, physicochemical-informed method selection, and integrated sensitivity analysis. The refined VU319 model provides a basis for evaluating formulation effects and informing the design of future multiple-ascending-dose studies, although its predictive performance under repeated-dose conditions remains to be established [9].

### 4.6. Limitations

This study evaluated a single compound, and broader applicability will require validation across multiple compounds. Although the model reproduced systemic exposure reasonably well, it did not fully reproduce the observed concentration–time profiles, particularly at higher doses. Formulation particle size was inferred from model calibration rather than measured experimentally, and biorelevant solubility characterization was limited to two pH conditions. BCS Class II designation was based on physicochemical properties and was not formally established. Parameter identifiability was assessed by varying one parameter at a time. Although the parameters showed distinct effects on model outputs, this approach does not exclude all alternative parameter combinations, and a joint evaluation of CL_int_ and particle radius would provide a more complete assessment. Dissolution-limited absorption is supported by measured solubility and observed exposure but has not been demonstrated experimentally. Biorelevant dissolution testing across the dose range and measurement of the formulation particle size distribution would be needed for confirmation. The supersaturation analysis relied on equilibrium solubility from biorelevant media, predicted bile micelle solubilization, and first-order precipitation with a default PRC, and it assumed a crystalline drug without excipients that promote supersaturation. Because luminal concentrations were not measured, in vivo supersaturation cannot be excluded. Gastric emptying, intestinal transit, and bile micelle solubilization were held at fasted-state defaults that did not vary with dose, and the back-calculated fraction absorbed assumes dose-independent clearance. Simulated prediction intervals were based on one virtual trial of six subjects per dose, matching the clinical cohort size, and therefore provide only an approximate estimate of population variability.

### 4.7. Future Directions

Direct measurement of formulation particle size distribution and biorelevant solubility in FeSSIF would help validate the dissolution-limited absorption hypothesis and strengthen confidence in the refined model. Measurement of hepatocyte intrinsic clearance would directly test the HLM/HH disconnect interpretation underlying the 10.7-fold CL_int_ correction.

Biorelevant solubility and dissolution testing at high dose loads would help determine whether prolonged or enhanced absorption explains the high-dose exposure. Characterization of CYP3A4 kinetic parameters (V_max_ and K_m_) would allow any contribution of saturable metabolism to be evaluated directly. The refined model also supports future applications in multiple-ascending-dose study design and formulation development for the VU319 program. In addition, the workflow can be evaluated for broader applicability using other low-solubility compounds with available SAD datasets.

## 5. Conclusions

PBPK modeling provided a structured framework for interpreting the divergence between IVIVE-based predictions and observed SAD pharmacokinetics for VU319. Sequential parameter refinement identified dissolution-limited absorption, represented through monodispersed particle radius, as the most likely contributor to the IVIVE–observed discrepancy. Secondary contributions arose from clearance, reflected by a downward CL_int_ correction consistent with an HLM/HH disconnect, and from distribution, reflected by K_p_ scaling of Poulin & Theil predictions.

The final model met predefined performance thresholds across the independent verification doses, while showing progressive underprediction at the highest doses (400 and 600 mg), for which a limited set of mechanistically plausible explanations was identified. Meeting these thresholds shows that the model reproduces observed systemic exposure, but it does not by itself confirm the underlying mechanism. This refinement workflow may be applicable to other low-solubility compounds in which standard IVIVE assumptions fail to adequately capture in vivo behavior. The model provides a basis for evaluating multiple-ascending-dose scenarios and formulation development, although prospective predictive performance remains to be established.

The mechanistic explanations identified in this study remain model-supported hypotheses that require independent experimental confirmation, including measurement of the formulation particle size distribution, biorelevant solubility and dissolution at high dose loads, hepatocyte intrinsic clearance, and CYP3A4 kinetic parameters.

## Figures and Tables

**Table 1:** Physicochemical and in vitro ADME inputs for VU319.

Parameter	Value	Unit	Source/Method
Physicochemical Properties			
Molecular weight	390.13	g/mol	In-house [10]
logP (octanol:water)	4.18	—	In-house [10]
logD_7_._4_	3.28	—	In-house [10]
Compound type	Monoprotic base	—	Structural assignment
pKa (conjugate acid)	1.4	—	In-house [10]
Fraction ionized at pH 7.4	<0.001%	—	Henderson–Hasselbalch
Hydrogen bond acceptors/donors	5/0	—	In-house [10]
Topological polar surface area	51	Å^2^	In-house [10]
Absorption			
Intrinsic solubility (S_0_)	0.0040852	mg/mL	Predicted (Simcyp Toolbox V24)
pH-solubility anchor 1 (pH 1.6)	0.05	mg/mL	Measured FaSSGF 24 h
pH-solubility anchor 2 (pH 6.5)	0.00532	mg/mL	Measured FaSSIF 24 h
Apparent permeability (Pₐ_pp_)	21.3 × 10^−6^	cm/s	In-house [10]
Particle dispersion type	Monodispersed	—	Default (Simcyp)
Particle radius (calibrated)	1	µm	Calibrated (Section 3.4)
Particle density	1.2	g/mL	Default (Simcyp)
PRC (precipitation rate constant)	4	h^−1^	Default (Simcyp)
CSR (critical supersaturation ratio)	10	—	Default (Simcyp)
Distribution			
Blood-to-plasma ratio (B/P)	1.29	—	In-house [10]
Fraction unbound (*f*_u_)	0.034	—	In-house [10]
V_ss_ prediction method	Method 1 (Poulin & Theil)	—	Selected per Section 2.6.1 [6]
V_ss_ (composition-based, uncalibrated)	7.895	L/kg	Predicted (Method 1, K_p_ scalar = 1)
Global K_p_ scalar (calibrated)	0.125	—	Calibrated (Section 3.2)
Lipid binding scalar	1	—	Default (Simcyp)
Metabolism			
Clearance model	Enzyme kinetics (CYP3A4)	—	In-house [10]
Microsomal protein concentration (incubation)	0.5	mg/mL	As used in HLM CL_int_ assay [10]
Fraction unbound in microsomes (*f*_u,mic_)	0.41286	—	Predicted (Hallifax–Houston at 0.5 mg/mL)
CYP3A4 CL_int_ (HLM, measured apparent)	32.2	µL/min/mg protein	In-house [10]
CYP3A4 CL_int_ (final, calibrated)	3	µL/min/mg protein	Calibrated (Section 3.3)
Elimination			
Renal clearance (CL_R_)	0	L/h	Conley et al. [11] (<0.1% recovered in urine)
Biliary CL_int_	0	µL/min/10^6^ cells	Default (no transporter-mediated efflux modelled)

**Table 2:** Local sensitivity of model outputs to each refined parameter, expressed as the fold-change in output across the evaluated input range.

Parameter	Evaluation Range	AUC_0–∞_ Fold-Change	C_max_ Fold-Change	Secondary Output (Fold-Change)
Particle radius ^1^	1–100 µm	3.9×	8.7×	T_max_, ~1.0 × (insensitive)
CYP3A4 CL_int_ ^2^	2–10 µL/min/mg	5.0×	1.1 × (insensitive)	t½, 4.8 × lower
Global K_p_ scalar ^3^	0.05–0.25	1.0 × (none)	2.3×	V_ss_, 4.3 × higher; t½, 4.4 × higher

^1^ Particle radius evaluated with CL_int_ fixed at the calibrated value of 3 µL/min/mg protein and K_p_ scalar fixed at 0.125. ^2^ CL_int_ evaluated with K_p_ scalar fixed at 0.125 and particle radius at 10 µm (Simcyp default). ^3^ K_p_ scalar evaluated with CL_int_ at the in vitro HLM value of 32.2 µL/min/mg protein (pre-clearance calibration) and particle radius at 10 µm. AUC_0–∞_ is governed by F × Dose/CL and is independent of tissue distribution at steady state; the primary sensitive outputs are V_ss_ and t_½_.

**Table 3:** Cohort-matched integrated model performance across the SAD range. Model values are geometric mean AUC_0–∞_ and arithmetic mean C_max_ from cohort-matched virtual populations (*n* = 6 per dose).

Dose (mg)	Observed AUC_0–∞_ (µg·h/mL)	Model AUC_0–∞_ (µg·h/mL)	AUC Fold (Model/Obs)	Observed C_max_ (µg/mL)	Model C_max_ (µg/mL)	C_max_ Fold
60	39.2	41.6	1.06	0.725	0.662	0.91
120 ^1^	59.0	62.3	1.06	0.884	0.794	0.90
240	78.3	83.1	1.06	1.18	0.934	0.79
400	124.0	78.8	0.64	1.72	0.995	0.58
600	221.0	89.7	0.41	2.81	1.127	0.40

^1^ The 120 mg cohort was the calibration reference; the 60, 240, 400, and 600 mg cohorts were not used for refinement.

**Table 4:** Dose number and dose-normalized exposure across the SAD range.

Dose (mg)	Do (FaSSIF)	Do (FaSSGF)	Observed AUC_0–∞_ (µg·h/mL)	Dose-Normalized AUC_0–∞_ (µg·h/mL per mg)
60	45	4.8	39.2	0.65
120	90	9.6	59.0	0.49
240	180	19	78.3	0.33
400	301	32	124	0.31
600	451	48	221	0.37

## Data Availability

Source clinical pharmacokinetic data are available in the original publication [11]. Simulation input files and complete model outputs are provided in the Supplementary Materials. Simcyp Simulator V24 (Certara) is a commercial product available under license.

## Supplementary Materials

The following supporting information can be downloaded at: https://www.mdpi.com/article/10.3390/pharmaceutics18101254/s1.

## Abbreviations

The following abbreviations are used in this manuscript:

ADAM	Advanced Dissolution, Absorption and Metabolism
ADME	Absorption, Distribution, Metabolism, and Excretion
API	Active Pharmaceutical Ingredient
AUC	Area Under the Plasma Concentration–Time Curve
AUC_0–∞_	Area Under the Plasma Concentration–Time Curve from Time Zero Extrapolated to Infinity
AUC_0–last_	Area Under the Plasma Concentration–Time Curve from Time Zero to the Last Measurable Concentration
B/P	Blood-to-Plasma Ratio
BCS	Biopharmaceutics Classification System
CL	Clearance
CL/F	Apparent Oral Clearance
CL_int_	Intrinsic Clearance
CL_R_	Renal Clearance
C_max_	Maximum Plasma Concentration
CSR	Critical Supersaturation Ratio
C_s_	Solubility (mg/mL)
CYP3A4	Cytochrome P450 3A4
Do	Dose Number
ECCS	Extended Clearance Classification System
FaSSGF	Fasted-State Simulated Gastric Fluid
FaSSIF	Fasted-State Simulated Intestinal Fluid
F	Oral Bioavailability
FDA	U.S. Food and Drug Administration
*F_g_*	Gut Bioavailability
*F_h_*	Hepatic Bioavailability
*f*_u_	Fraction Unbound in Plasma
*f*_u,b_	Fraction unbound in blood
*f*_u,mic_	Fraction Unbound in Microsomal Incubation
GMFE	Geometric Mean Fold Error
HLM	Human Liver Microsomes
HH	Hepatocytes
IND	Investigational New Drug
IR:DLM	Immediate-Release Diffusion Layer Model
IVIVE	In Vitro–In Vivo Extrapolation
K_m_	Michaelis Constant
K_p_	Tissue-to-Plasma Partition Coefficient
M_0_	Administered Dose (mg)
PBBM	Physiologically Based Biopharmaceutics Modeling
PBPK	Physiologically Based Pharmacokinetic
P_app_	Apparent Permeability
P_eff_	Effective Permeability
PK	Pharmacokinetic
PO	Oral
PRC	Precipitation Rate Constant
SAD	Single Ascending Dose
SD	Standard Deviation
Simcyp	Simcyp Simulator
SR	Supersaturation Ratio
t½	Terminal Elimination Half-Life
T_max_	Time to Maximum Plasma Concentration
V_0_	Fluid Volume Taken with Dose
V_ss_	Volume of Distribution at Steady State
V_z_/F	Apparent Volume of Distribution During the Terminal Phase
